# A Crusade against Indifference

**Published:** 1887-06-18

**Authors:** 


### A Crusade against Indifference.

Hospitals are not perhaps what, in popular parlance, may be termed a sensational topic. In these days of multitudinous meetings, unless the topic is sensational —one that for a time enchains the interest of the town —there is not much chance of filling big halls, especially in the middle of June. Despite the fact that we live in what has been termed "an age of philanthropy," there is a vast barrier of public apathy which must, somehow or another, be broken down. We can no longer leave the support of our hospitals to the rich alone, nor is it just that we should do so. Though they exist for the good of all classes, it is the poor whom they primarily benefit, and the time has now imperatively come when the poor man's penny must no longer be withheld. The great object in view in the establishment of this Journal was to arouse a wider and a deeper popular interest in our hospitals, for it was felt that a crisis must inevitably come, unless a general awakening of the public conscience to the grand work of medical charities, and to their urgent appeals for help, could be accomplished. Journalism has done much to educate the minds of the masses, but unfortunately the secular press has not generally recognised its duty in respect to the hospitals. If it had done more to arouse the minds of the people to the fact that hospitals exist for the common good, that we are greatly indebted to them for the healthfulness of our great cities, and that their existence confers a material benefit upon one and all, the present sad plight of medical charities might have been altogether avoided. We have ourselves endeavoured to do what has been too long neglected, and are happy to know that the task we set ourselves to do has by no means been fruitless of a good result. "Light, more light," were the last words of the dying Goethe. "Light, more light," is just now the pressing need of the people of this country in reference to the magnificent work which our hospitals are doing. When this is given to the people, we have no fear that the generous hearts of Englishmen will fail to respond liberally to the call of charity. At present we must work assiduously to give this light, and to carry on the crusade against indifference. The thin attendances at most of the meetings which have been held during the Hospitals Fortnight have amply shown the indifference which exists, but which we hope and believe the persistent efforts of the charitable among us will speedily remove.

### MEETING AT HAMPSTEAD.

The third meeting in connection with the Hospitals Fortnight was held at the Hampstead Vestry Hall, under the presidency of Lord William Compton, the attendance numbering only some seventy or eighty. Among others present were Sir Sydney Waterlow, Sir Joseph Fayrer, Dr. Kennedy, the Rev. S. B. Burnaby (vicar of Hampstead), the Chevalier Jno. Ortelli, the munificent supporter of the Italian Hospital, Rev. R. F. Horton, M.A., Rev. Edward Bond, M.A., Mr. Miller, and Mr. Oakley, the hon. secretary. Among others who were unable to be present were the Marquis of Lorne, Lord Cranborne, Lord Robartes, Dr. Cooper Rose, Sir W. Onslow, Sir Richard Temple, Sir Edmund Hay Currie, and Sir Henry and Lady Holland.

The Chairman, after explaining the objects of the meeting, observed, alluding to the limited attendance, that if they had not a sensational subject to attract, they had at least one which called forth greater sympathy from unselfish, God-fearing men and women. Whatever might happen, we must always have the poor and the sick amongst us. Accidents must happen, sicknesses must occur, and for these we must provide and give the aid that was necessary. Though doubtless most of us had been asked to subscribe to national and local schemes for the Jubilee celebration, the pockets of the people were surely not so empty that they could not afford to help the Hospital Sunday Fund. After quoting the statistics issued by the Mansion House Committee showing the huge need for the efficient maintenance of our medical charities, his lordship concluded by expressing the hope that the people of England would not allow a national disgrace to come upon them through their neglect of a public duty.

The Rev. S. B. Burnaby, in moving the first resolution—which has been of a stereotyped character at all the meetings—remarked that this was an age of meetings, but gatherings such as theirs, called for purely benevolent and merciful purposes, had a peculiar significance. It was universally acknowledged that the hospitals were carrying on a most noble work in alleviating suffering, in explaining and enforcing the laws of health, but there was another aspect of their work which, although of vast importance to the people, had not been much considered, viz., the moral influence which hospitals exercised, a fact which might be learnt from the pages of The Hospital. There could, he thought, be few patients who left a hospital without a deep sense of gratitude for the benefits they had received; some under their affliction learn wisdom, others to appreciate the generous self-sacrifice made on their behalf. There were few who left the hospital without in some way becoming more moral, better men and women, better fathers and mothers, with a clearer understanding of the duties and relationships of life. The hospitals were thus a benefit, not only to individuals, but to society at large, and to the State generally. They proved, that those who were highest in intelligence did care for the wants of the people, and generally endeavoured to minister to those wants. Touching on the educational aspect of hospitals, the vicar contended that there were, and could be, no classrooms like the bedsides of the wards, where every symptom was watched and registered, and the information stored up with an exactitude which was impossible in private practice. All those who desired to have in the future a wise and skilled medical profession, as well as all those who desired to alleviate suffering, were debtors to the hospitals. If we failed to take sufficiently to heart our obligations in both these respects, we should have to pay as ratepayers what we had refused to give in charity and kindness.

The speech of Sir Sydney Waterlow, who seconded the resolution, will be found in another portion of our impression.

Sir Joseph Fayrer moved the second resolution: "That this meeting regrets to learn that the expenditure and liabilities of the hospitals and medical charities in this district in 1886 exceeded their income by £8,000, and it pledges itself to increased exertion with a view to preventing any like deficiency in the current year." He was afraid that many gave exceedingly little. If the people would only increase their subscriptions a little, we should not have the same story to tell about every hospital exceeding its income. Our hospitals and other charitable institutions in London, and in the United Kingdom generally, were really a source of national gratification and pride, because in no other country of the world were so many noble charities supported by voluntary contributions. It was quite clear that the maintenance of our hospitals was a duty which devolved upon all. We were bound to it in the interest of the poor, the sick, and the wounded, who must be cared for, but who could not care for themselves. Sir Joseph next referred in eloquent terms to the magnificent work which the Italian Hospital in London was carrying on, founded by the munificence of the Chevalier Ortelli. The institution was as yet only three years old, and for this reason it had not hitherto been able to participate in the Hospital Sunday Fund. It was thoroughly entitled to our sympathy and support, and he was glad to have been able to take that opportunity to urge its claim upon the Fund.

The Rev. R. F. Horton, M.A., in seconding the resolution, confessed that his sympathies lay rather with the provident dispensaries than with the hospitals, on the principle that we should try to help those who tried to help themselves.

Mr. Miller, speaking in support of the resolution, said he would advance two reasons why we should support our medical charities: if we had had sickness ourselves or in our families during the past year, we should subscribe out of sympathy with our fellow-sufferers; if we had not, then we should subscribe largely as a thank-offering to God for sparing us.

Rev. Dr. Kennedy, who has been connected with the Hospital Sunday Fund from its beginning, spoke of the eminent services which Sir Sydney Waterlow had rendered to it, and detailed the scheme which governs the working and organisation of the Fund, and referred to its thoroughly democratic character. Dr. Kennedy concluded with a vote of thanks to the chairman, seconded by Mr. Edward Bond. Lord William Compton briefly replied, and the proceedings terminated.

